# Taking publication date into consideration: a response to the review of *Mobile Technologies for Every Library*

**DOI:** 10.5195/jmla.2018.397

**Published:** 2018-04-01

**Authors:** Lisa Mastin

**Affiliations:** Medical Librarian, Fay E. Evatt Medical Library, WellStar Atlanta Medical Center, Atlanta, GA

**To the editor,** I am writing in response to the recent review of Gleason’s *Mobile Technologies for Every Library* [1]. I agree that it is difficult to remain current when writing about technology. I also appreciate the assertion made by the author of the review, who felt that the book’s shortcomings were its timeliness and lack of insight into emerging technologies, that a review is obliged to provide supporting evidence for its judgments.

However, I have concerns surrounding the arguments offered as proof that the book is largely outdated and an impractical choice for many libraries. My first issue is with the labeling of the data in the book (i.e., 2014 Horizon report, 2013 Smartphone Market Shares) as woefully out of date. The book was published in 2015; therefore, data from 2013 and 2014 seem appropriate and likely were the most current data available.

Secondly, the labeling, by the review’s author, of Figure 4.2 in the book as laughable because the listed Android versions were not the most recent seems unfair. Android versions Lollipop, Marshmallow, and Nougat were released in 2014, 2015, and 2016, respectively [2]. Gleason could not be expected to include yet-to-be released Android versions in her book.

Finally, I come to the defense of the book’s author for her choice to include Google Glass as a relevant mobile technology, which the author of the review criticized as “not new and exciting.” For some libraries, Google Glass, as well as other technologies mentioned in the book (i.e., virtual reality, augmented reality), would be considered new and possibly exciting because many libraries have limited technologies due to demographics, budgets, and library size. Furthermore, Rawlins’s book that the reviewer recommended also mentions Google Glass among its referenced technologies.

A review may be obliged to offer supporting evidence for its criticisms, but more importantly, it must take into account the publication date of the book being reviewed. It is worth noting that although technology may quickly become obsolete, not all libraries can keep up with the pace.
